# Pseudoarthrogram sign — a rare radiological appearance of implant failure in the knee 

**DOI:** 10.1093/bjrcr/uaae033

**Published:** 2024-10-11

**Authors:** Bilal A Khan, Gorav Datta, Neeraj Purohit

**Affiliations:** Radiology Department, University Hospital Southampton, Southampton, Hampshire SO166YD, United Kingdom; Radiology Department, University Hospital Southampton, Southampton, Hampshire SO166YD, United Kingdom; Radiology Department, University Hospital Southampton, Southampton, Hampshire SO166YD, United Kingdom

**Keywords:** oxinium, arthrogram, knee arthroplasty

## Abstract

We present the radiological findings in the case of a failed unicompartmental knee arthroplasty (UKA). Although uncommon, these features are highly specific for implant failure and are an indication to consider revision. The aim of this case report is to highlight these characteristic appearances across multiple imaging modalities to both surgeons and radiologists should they encounter this in their practise.

## Background

Primary knee arthroplasty remains one of the most frequently performed surgical procedures with approximately 90 000 operations carried out every year in the United Kingdom.^1^ Common complications encountered radiologically include infection, peri-prosthetic fractures, dislocations, heterotypic ossification, and aseptic loosening.^2^^,^^3^

Adverse reaction to metal debris (ARMD) or metallosis is a rare complication after arthroplasty mostly frequently described with metal-on metal prosthesis following total hip replacement.^4^ This is however less frequently described in the knee, particularly with the use of newer implant materials such as oxidized zirconium (oxinium) initially introduced as an alternative bearing surface to reduce polyethylene wear and aseptic loosening in knee arthroplasty.^5^

We present a case of a failed unicompartmental knee arthroplasty (UKA) resulting in articulation of an oxidized zirconium femoral component against the tibial component with severe polyethylene wear-through and disassociation. This resulted in widespread fine synovial deposits of oxidized zirconium causing a characteristic “pseudoarthrogram” or “oxinium arthrogram” sign on plain radiograph.

The aim of this case report is to highlight the characteristic appearance of the pseudoarthrogram sign across multiple imaging modalities to both surgeons and radiologists should they encounter this in their practise.

## Case report

A 71-year-old male with no other past medical history presented to the clinic with left knee swelling and pain which began following a fall that occurred 6 weeks after a UKA for medial compartment arthritis. Clinical examination revealed a small joint effusion and a well-healed surgical incision without evidence of erythema. Range of movement from 0° to 90° was noted with no significant laxity to varus or vagus stress. Joint aspiration revealed a dark-coloured fluid aspirate with no evidence of leucocytosis or growth on culture. Operative records confirmed the use of an oxinium femoral component.

**Figure 1.**
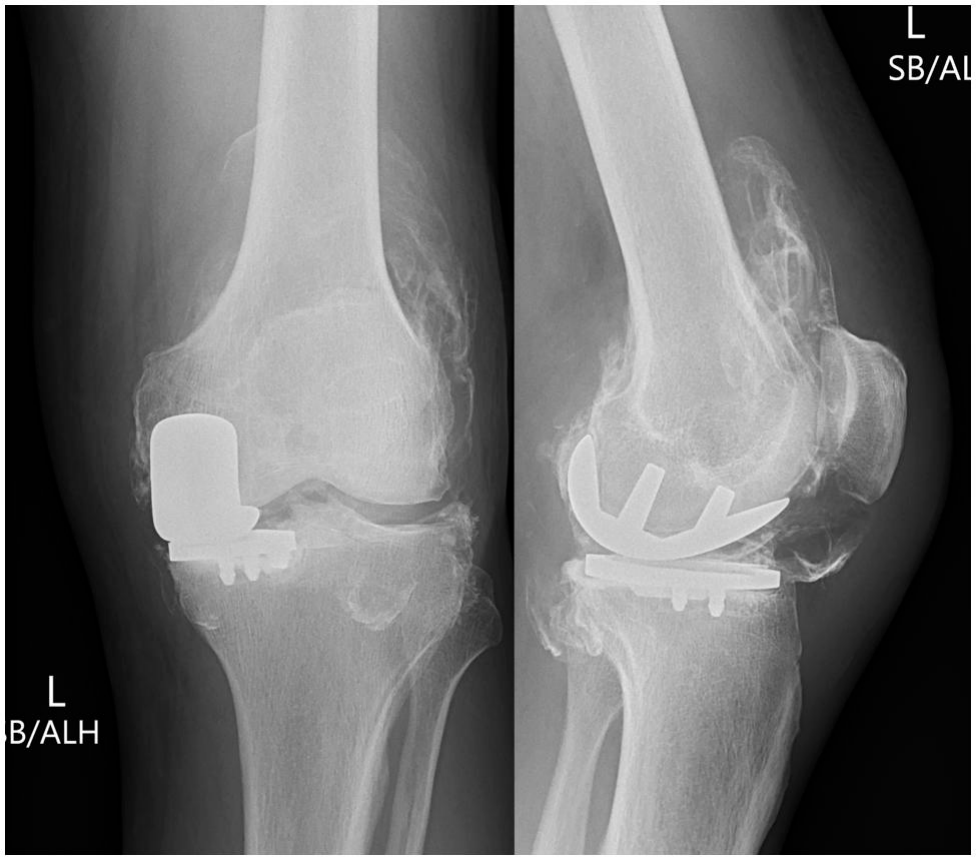
Frontal and lateral radiograph of the left knee. This shows a medial compartment knee arthroplasty with loss of the expected space between the femoral and tibial components. There is also fine high-density material outlining the joint capsule.

**Figure 2.**
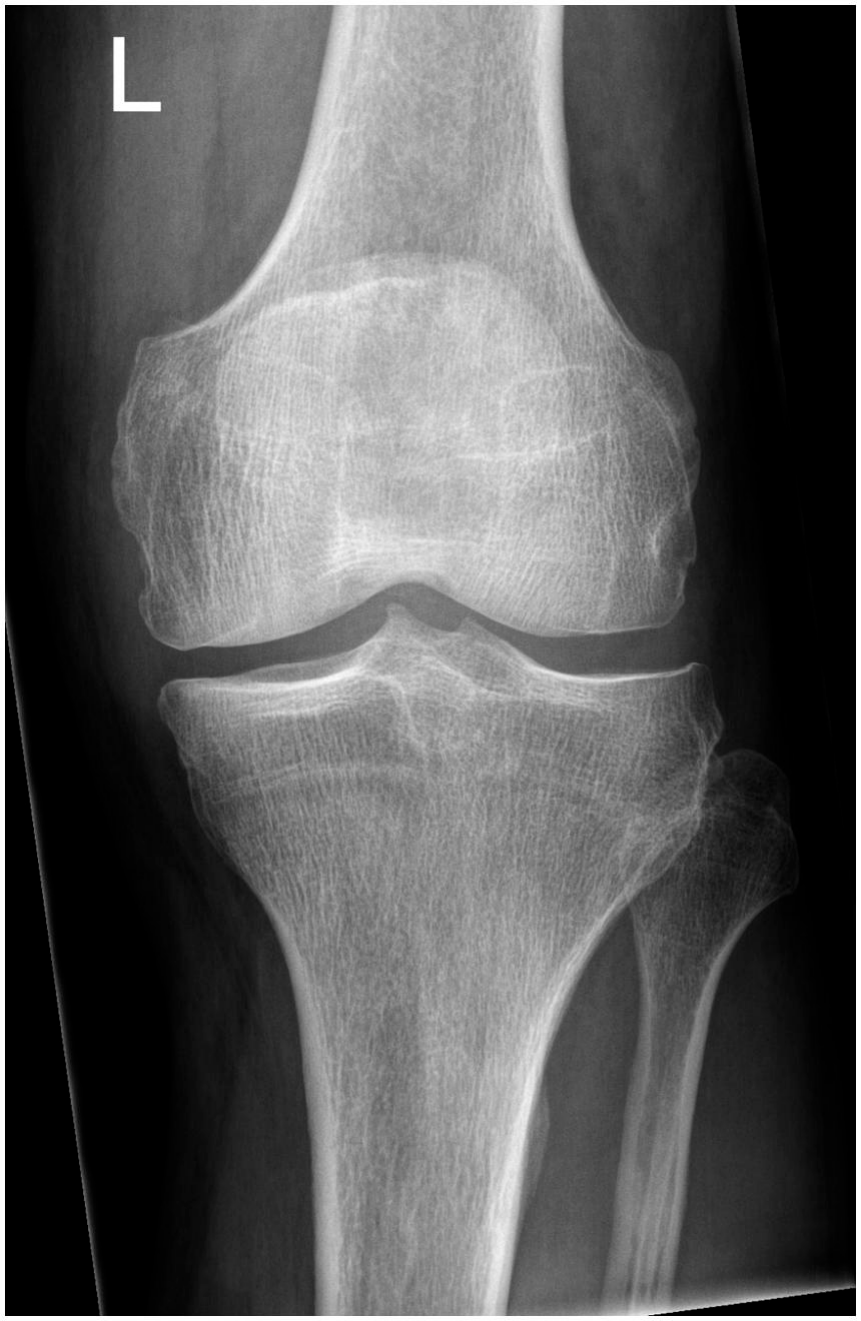
Frontal radiograph of the left knee. This shows the pre-operative appearance of the knee highlighting no pre-existing abnormality of the joint capsule.

**Figure 3.**
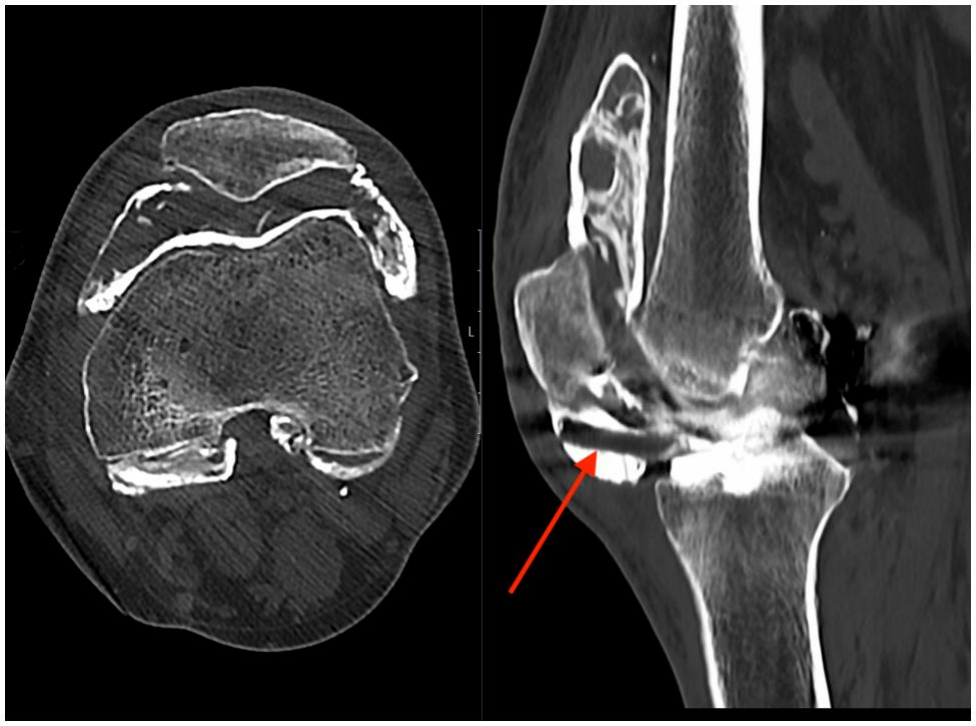
Axial and sagittal CT images of the left knee. These images further highlight the high-density material outlining the joint capsule producing a “pseudo-arthrogram” appearance. Additional note made is also made of an anteriorly displaced polyethylene liner (arrow).

Initial radiographs revealed fine high-density debris in the supra-patellar, infra-patellar, and posterior joint recesses (Figure 1). There also apparent loss of the normal radiolucency between the femoral and tibial components normally produced by the polyethylene liner. A preoperative radiograph shows no such pre-existing abnormality (Figure 2). A subsequent CT scan shows thick, irregular high-density material depositing in the synovial lining outlining the joint capsule resulting in a pseudo-arthrogram sign (Figure 3). The sagittal image highlights a well-demarcated radiolucency communicating with the medial compartment which protrudes anteriorly into the infra-patellar region (arrow). This is the characteristic appearance of a dissociated polyethylene liner.

**Figure 4.**
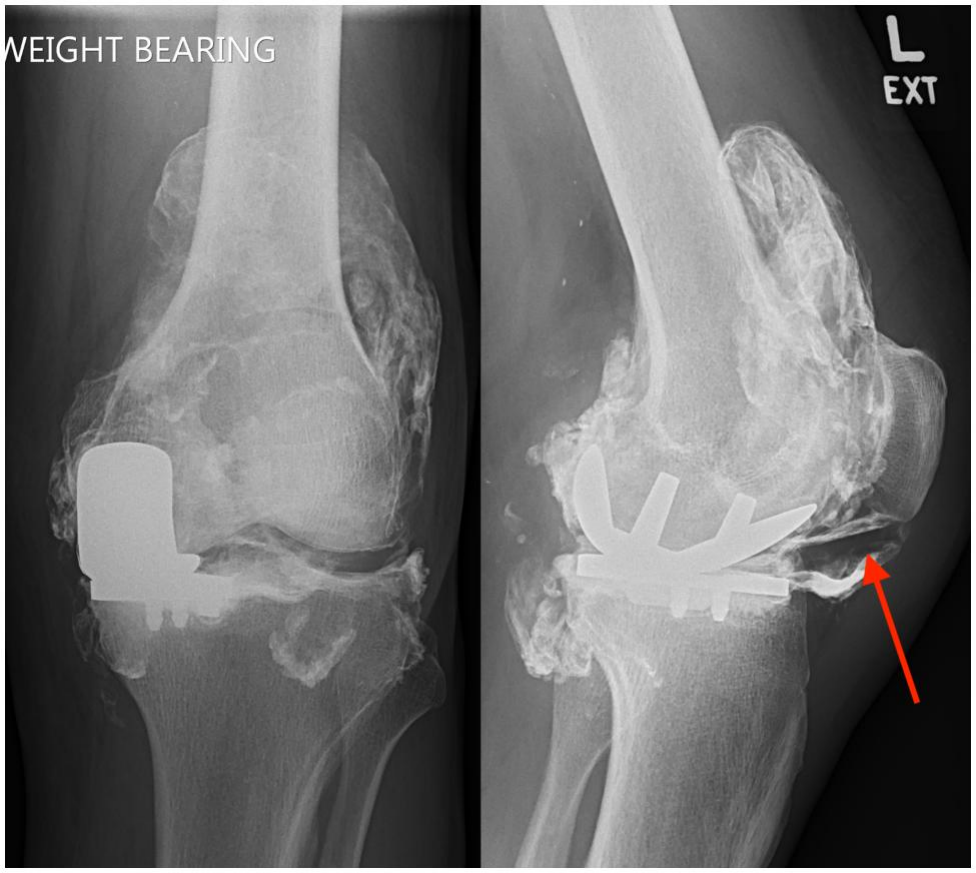
Frontal and lateral radiograph of the left knee. This shows progressive disposition of the high-density material on the joint capsule which results is appreciation of the displaced polyethylene liner (arrow).

The patient was listed for revision surgery due to complete failure of UKA with displacement of the polyethylene liner. This resulted in contacting of the oxinium femoral component against the tibial prosthesis leading to the subsequent pseudo-arthrogram radiographic appearance. A preoperative radiograph performed 4 weeks from the initial radiograph showed progressive dense synovial deposits which allowed visualization of the anteriorly displaced polyethylene liner (Figure 4).

**Figure 5.**
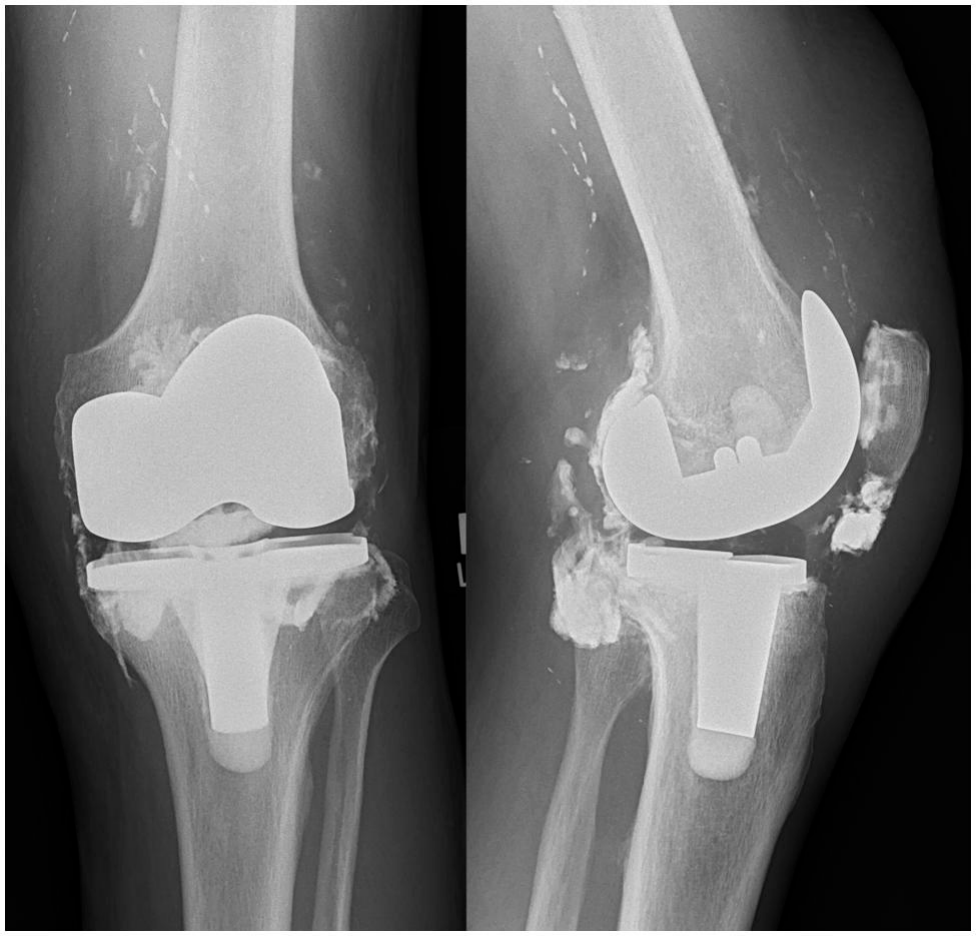
Frontal and lateral radiograph of the left knee. This shows revision to total knee replacement with synovectomy resulting in markedly improved appearances of the joint capsule.

Intraoperative findings included extensive oxinium deposits on the synovial lining of knee joint correlating to the radiographic findings with associated black-coloured joint fluid. Prominent oxinium deposits were also seen coating the native lateral and patello-femoral articular surfaces of knee. The tibial baseplate was also noted to be loose although there was no radiological evidence of this pre-operatively. The patient underwent extensive synovectomy and single-stage revision to total knee replacement (Figure 5). The patient’s post-operative rehabilitation was successful with marked improvement in symptoms and functionality at 1 year.

## Discussion

This case report highlights the rare but very characteristic radiographic appearance of a pseudoarthrogram sign which can be easily misinterpreted if not previously encountered. In this case the pseudoarthrogram appearance was the result of polyethylene liner dislocation in a UKA resulting in direct articulation of the oxidized zirconium femoral component with the tibial baseplate. Intraoperative findings confirmed widespread oxinium deposits within the synovium and native articular surfaces.

Greco and Berend have described the only other published case of UKA failure with an oxinium prosthesis leading to the pseudo-arthrogram sign.^6^ This case differed slightly in that failure of the UKA occurred at only 6 weeks post-operatively (vs 15 months in Greco and Berend) with significant tibial component loosening also seen in our case. Our case also clearly highlights the additional finding of the displaced polyethylene liner both radigraphically and on CT. A few case reports have also described failure of oxinium total knee arthroplasty (TKA) leading to a pseudoarthrogram appearance on plain radiographs only.^7^^,^^8^ Two similar cases by Gkouliopoulou et al^9^ (Polyethylene wear-through and mechanical loosening) and Tribe et al^10^ (Polyethylene dissociation) have described an oxinium pseudo-arthogram appearance following failed total hip arthroplasty.

While the appearance of a pseudoarthrogram is relatively uncommon, the clinical implications are significant with all highlighted cases requiring revision. Hence prompt recognition and accurate diagnosis is crucial to patient management. By highlighting this case with classic findings across multiple modalities we aim to increase awareness amongst surgeons and radiologists in order to promote accurate diagnosis.

## Learning points

- Be able to appreciate the characteristic pseudoarthrogram/oxinium arthrogram appearances on plain radiographs which indicate implant failure
- Be able to recognize the appearance of a displaced polyethylene liner on radiographs and CT.
- Be aware of this potential complication when encountering oxinium implants.

## References

[1] Ben-Shlomo Y , Blom A, Boulton C, et al The National Joint Registry 19th Annual Report. 2022. Accessed September 6, 2024. https://europepmc.org/article/med/33439585. 36516281

[2] Roth TD , Maertz NA, Parr JA, Buckwalter KA, Choplin RH. CT of the hip prosthesis: appearance of components, fixation, and complications. Radiographics. 2012;32(4):1089-1107. 22786996 10.1148/rg.324115183

[3] Chang EY , McAnally JL, Van Horne JR, et al Metal-onmetal total hip arthroplasty: do symptoms correlate with MR imaging findings? Radiology. 2012;265(3):848-857. 23047842 10.1148/radiol.12120852

[4] Chalmers BP , Perry KI, Taunton MJ, Mabry TM, Abdel MP. Diagnosis of adverse local tissue reactions following metal-on-metal hip arthroplasty. Curr Rev Musculoskelet Med. 2016;9(1):67-74. 26816329 10.1007/s12178-016-9321-3 PMC4762796

[5] Civinini R , Matassi F, Carulli C, Sirleo L, Lepri AC, Innocenti M. Clinical results of oxidized zirconium femoral component in TKA. A review of long-term survival. HSS J. 2017;13(1):32-34. 28167871 10.1007/s11420-016-9512-x PMC5264569

[6] Greco N , Berend K. Polyethylene liner dislocation of fixed-bearing medial oxinium unicompartmental arthroplasty with severe metallosis. Knee. 2018;25(2):341-345. 29525546 10.1016/j.knee.2018.01.004

[7] Frye BM , Laughery KR, Klein AE. The oxinium arthrogram: a sign of oxidized zirconium implant failure. Arthroplast Today. 2021;8:103-109. 33732834 10.1016/j.artd.2021.02.001 PMC7943965

[8] Kore L , Bates T, Mills G, Lybeck D. Oxidized zirconium total knee arthroplasty implant failure in a patient with knee instability. Arthroplast Today. 2020;6(3):552-555. 32775586 10.1016/j.artd.2020.06.015 PMC7397700

[9] Gkouliopoulou E , Agathangelidis F, Vampertzis T, Ntovas T. Severe metallosis following oxidised zirconium wear in total hip arthroplasty. BMJ Case Rep. 2016;2016:bcr2016218025. 10.1136/bcr-2016-218025 PMC5093348 27799229

[10] Tribe H , Malek S, Stammers J, Ranawat V, Skinner JA. Advanced wear of an Oxinium™ femoral head implant following polyethylene liner dislocation. Ann R Coll Surg Engl. 2013;95(8):e4-e6. 10.1308/003588413X13629960047876 PMC4311548 24165329

